# Detection of Brief Episodes of Atrial Fibrillation Based on Electrocardiomatrix and Convolutional Neural Network

**DOI:** 10.3389/fphys.2021.673819

**Published:** 2021-08-25

**Authors:** Ricardo Salinas-Martínez, Johannes de Bie, Nicoletta Marzocchi, Frida Sandberg

**Affiliations:** ^1^Mortara Instrument Europe s.r.l., Bologna, Italy; ^2^Department of Biomedical Engineering, Lund University, Lund, Sweden

**Keywords:** atrial fibrillation, brief atrial fibrillation, convolutional neural network, interpretability, atrial fibrillation detection, layer-wise relevance propagation, long-term ECG

## Abstract

**Background:** Brief episodes of atrial fibrillation (AF) may evolve into longer AF episodes increasing the chances of thrombus formation, stroke, and death. Classical methods for AF detection investigate rhythm irregularity or P-wave absence in the ECG, while deep learning approaches profit from the availability of annotated ECG databases to learn discriminatory features linked to different diagnosis. However, some deep learning approaches do not provide analysis of the features used for classification. This paper introduces a convolutional neural network (CNN) approach for automatic detection of brief AF episodes based on electrocardiomatrix-images (ECM-images) aiming to link deep learning to features with clinical meaning.

**Materials and Methods:** The CNN is trained using two databases: the Long-Term Atrial Fibrillation and the MIT-BIH Normal Sinus Rhythm, and tested on three databases: the MIT-BIH Atrial Fibrillation, the MIT-BIH Arrhythmia, and the Monzino-AF. Detection of AF is done using a sliding window of 10 beats plus 3 s. Performance is quantified using both standard classification metrics and the EC57 standard for arrhythmia detection. Layer-wise relevance propagation analysis was applied to link the decisions made by the CNN to clinical characteristics in the ECG.

**Results:** For all three testing databases, episode sensitivity was greater than 80.22, 89.66, and 97.45% for AF episodes shorter than 15, 30 s, and for all episodes, respectively.

**Conclusions:** Rhythm and morphological characteristics of the electrocardiogram can be learned by a CNN from ECM-images for the detection of brief episodes of AF.

## 1. Introduction

Atrial fibrillation (AF) is the most common heart rhythm disorder found in clinical practice, and it is highly correlated with stroke (Wolf et al., 1991). Guidelines for the management of AF episodes lasting more than 30 s are available (Hindricks et al., 2020). However, less information is known for episodes shorter than 30 s, frequently known as brief AF. Atrial fibrillation is a progressive arrhythmia for which even brief episodes could represent a risk factor for thrombus formation and stroke (Healey et al., 2012). Additionally, in case of delayed management of such brief episodes, they may evolve into longer AF episodes (Hindricks et al., 2020). For this reason, it is a clinical recommendation to monitor stroke patients with long-term ECG recordings to assess the presence of brief AF episodes (Hindricks et al., 2020). However, manual revision of large amounts of ECG data is highly time-consuming, might be influenced by reviewers' subjectivity, and represents a significant financial burden in healthcare (Lee et al., 2008; Kim et al., 2011; Ball et al., 2013). To avoid these problems, different approaches for AF detection have been proposed based on ECG rhythm and morphology analysis, compact representation of the ECG for visual inspection, and deep learning (DL) techniques (Hagiwara et al., 2018; Rizwan et al., 2020).

Atrial fibrillation detectors based on rhythm information rely on different detection metrics. For instance, Zhou et al. (2015), Lake and Moorman (2011), and Petrėnas et al. (2015) developed methods based on entropy metrics; Lee et al. (2013) presented a linear system approach for AF detection by exploring the difference in spectral coherence of the RR intervals present in two continuous time windows; Huang et al. (2010) proposed an approach based on the histogram of the RR interval series. Nevertheless, some of these approaches require a data buffer of at least 1 min for adequate performance. Other authors have proposed to combine rhythm and morphological information as the detection window is reduced. For instance, de Carvalho et al. (2012) applied the spectral entropy for determining f wave presence; Babaeizadeh et al. (2009) investigated the absence of P waves by measuring the similarity between the samples in two consecutive PR intervals. Conversely, Ladavich and Ghoraani (2015), Ródenas et al. (2015), and Ródenas et al. (2017) only explored whether P waves are absent and left out all rhythm information. However, the detectors accounting for morphological information have not been completely capable of characterizing the atrial activity, which is commonly obscured by noise, leading to similar performances as those accounting for rhythm (Sörnmo, 2018).

In terms of ECG visual inspection, Li et al. (2015) proposed the electrocardiomatrix (ECM) for visualization of long-term ECG recordings by presenting the information in a compact, two-dimensional, form while preserving morphology and rhythm characteristics. This approach is intended to simplify manual review of ECG recordings for AF detection and to reduce the workload for reviewers. Implementing this technique, Lee et al. (2018) concluded that the ECM technique is a reliable method for accurate detection of AF when ECM-images of long-term ECG recordings are analyzed by experienced ECG-reader. The detection is made after the alignment of detected R peaks. Such alignment facilitates evaluating whether or not they are preceded by P waves. Additionally, aligning the R peaks in the ECM enables detection of rhythm irregularity present in long-term recordings. However, it remains unclear whether ECM can be used for automatic detection of brief AF episodes.

Deep learning (DL) techniques can reduce the workload in decision-making tasks, leading to faster and more consistent decisions while releasing human resources for other tasks. In this sense, increased computational power and the availability of ECG databases with clinical annotations have driven the development of DL techniques for unsupervised ECG analysis (Parvaneh et al., 2019; Somani et al., 2021). For the detection of AF different DL methodologies have been proposed, including hierarchical attention networks (Mousavi et al., 2020), long short-term memory (Faust et al., 2018; Andersen et al., 2019; Dang et al., 2019; Jin et al., 2020), convolutional neural network (CNN) (He et al., 2018; Xia et al., 2018; Lai et al., 2019; Huang and Wu, 2020; Zhang et al., 2020), and approaches combining recurrent neural networks with CNN (Fujita and Cimr, 2019; Shi et al., 2020; Wang, 2020). Some of these approaches are trained with raw ECG signals (Dang et al., 2019; Huang and Wu, 2020; Jin et al., 2020; Mousavi et al., 2020; Shi et al., 2020; Wang, 2020), or with series of RR intervals (Faust et al., 2018; Andersen et al., 2019; Dang et al., 2019; Lai et al., 2019), while some others utilize time-frequency domain information extracted from the ECG. For the latter, different transformations have been used to create time-frequency images from the ECG such as the spectrogram (Xia et al., 2018), the scalogram (He et al., 2018; Jin et al., 2020), and the stationary wavelet transform (Xia et al., 2018; Zhang et al., 2020).

One drawback of some DL approaches for AF detection is that they are trained and tested with intra-patient subsets of the same database, increasing the chances of overfitting (He et al., 2018; Xia et al., 2018; Dang et al., 2019; Fujita and Cimr, 2019; Lai et al., 2019; Huang and Wu, 2020; Mousavi et al., 2020; Shi et al., 2020; Wang, 2020; Zhang et al., 2020). In this sense, further approaches should consider to train, validate, and test the hyperparameters in the network following a inter-patient approach, i.e., perform the validation and testing with records that have not been used during the training process.

Deep learning techniques have been frequently considered as “black boxes,” not providing information about the decision process. The lack of transparency and explainability could be addressed by investigating which part of the input data is more relevant for the classification. Such analysis could support the adequate functioning of the system (Montavon et al., 2018; Samek et al., 2019), and it could also help detecting biases in the model or data. In this context, Bach et al. (2015) proposed the layer-wise relevance propagation (LRP) technique to analyze the decisions made by deep neural networks. The LRP propagates the prediction score backward through the model using a set of local redistribution rules. This technique can be applied to DL systems providing interpretation with respect to the input (Binder et al., 2018). Layer-wise relevance propagation was applied in brain-computer imaging to analyze factors leading to low-confidence decisions made by a neural network (Sturm et al., 2016). Similarly, LRP has been applied to interpret the decisions of a non-linear machine learning method in biomechanical gait analysis (Horst et al., 2019), and therapy decisions in healthcare applications (Yang et al., 2018) providing insights on the overall decision process of DL systems. Such analysis technique could be applied to DL systems for AF detection as interpretability of these approaches is becoming critical in the medical context.

Despite the great advances in automatic ECG interpretation, detection of brief AF episodes in long-term ECG recordings remains an open challenge, both for classical and DL techniques, due to their paroxysmal and intermittent nature. Classical methods investigate rhythm irregularity or P-wave absence in the ECG, while DL approaches profit from the availability of annotated ECG databases to learn discriminatory features linked to different diagnoses. However, some DL approaches do not consider inter-patient evaluation, and are tested on a small subset of ECG records; increasing the chances of overfitting. Additionally, many of the DL approaches do not provide analysis of the features used for the classification. This paper investigates whether ECM-images and CNN can be used together for detecting brief episodes of AF. The study also investigates the influence of leads used for training as well as the performance on databases not used in the training process. Finally, we implemented the LRP technique to evaluate whether the network is capable of learning the well-known characteristics of AF from ECM-images.

## 2. Materials and Methods

This section provides a detailed specification of the databases utilized during the study as well as the methodology for the generation of the ECM-images. Next, a full description of the CNN architecture and the process for the LRP analysis is provided. Finally, the metrics used for evaluation are presented.

### 2.1. Databases

Four public databases available at PhysioNet (Goldberger et al., 2000), and one proprietary database are used in this study. All databases are provided with manual annotations for different rhythms following the standard syntaxis used by Physionet (Goldberger et al., 2000). A general description of the databases is presented in Table 1.

**Table 1:** General description of databases used in the study.

**Database**	**LTAFDB**	**NSRDB**	**AFDB**	**Arrhythmia DB**	**Monzino-AF DB**
Subjects	47	18	–	47	35
Records used (total)	84	18	23 (25)	48	38
Records with NSR	2	18	0	28	9
Records with AF	82	0	23	8	26
Records with other arrhythmias	0	0	0	12	3
Channels	2	2	2	2	9
Total duration	1,960 h	437 h	234 h	24 h	61 h
Number of ECMs in non-AF	768,124	347,670	60,866	9,495	6,689
Number of ECMs in AF	1,052,506	0	54,459	1,077	24,272
Utility	Training	Training	Testing	Testing	Testing

The Long-Term Atrial Fibrillation (LTAFDB) contains 84 two-lead long-term ECG records, lasting from 24 to 25 h and sampled at 128 Hz, from patients with paroxysmal or persistent AF (Petrutiu et al., 2007). The MIT-BIH Normal Sinus Rhythm (NSRDB) consist of 18 two-lead long-term ECG records, lasting from 23 to 26 h, and acquired with a sampling frequency of 128 Hz, from patients without any significant arrhythmia (Goldberger et al., 2000). The MIT-BIH Atrial Fibrillation (AFDB) is a set of 25 two-lead long-term ECG records lasting 10 h acquired with a sampling frequency of 250 Hz, from patients with AF, mostly paroxysmal, (Moody, 1983). The records 00735 and 03665 from the AFDB, including only rhythm information, are excluded in this study. The MIT-BIH Arrhythmia database (Arrhythmia DB) contains 48 two-lead records including a variety of rare but clinically important cardiac conditions (Moody and Mark, 2001). Each of the 48 records is slightly over 30 min long and was sampled at 360 Hz. The proprietary Monzino-AF database (Monzino-AF DB) includes 38 records from 35 patients, 12 lead telemetry monitoring ECG records with total database-duration of 61 h, sampled at a rate of 500 Hz. This database is useful to evaluate performance using different leads. The records were collected following the declaration of Helsinki, in the Ventricular Intensive Care (VIC) unit for the intensive treatment of ventricular arrhythmias (at Centro Cardiologico Monzino) specialized in the treatment of patients with heart disease with severe arrhythmias. Patients admitted to the VIC unit are usually treated with a transcatheter ablation to control their severe recurrent arrhythmias. This procedure is performed using special probes called electrocatheters or, less often, using a surgical approach. During hospitalization ECG of patients is continuously monitored either in the pre- and post- operative period. The records are manually annotated for AF by expert ECG-readers from Hillrom, and reviewed by clinical experts within the company, asking for advice to cardiologist whenever needed. The LTAFDB and the NSRDB are used for training and validation, whereas the AFDB, the Arrhythmia DB and the Monzino-AF DB are used for testing.

### 2.2. Electrocardiomatrix

The ECG signal is upsampled to 500 Hz using the *xform* tool from Physionet (Goldberger et al., 2000). The upsampling is needed to fulfill the requirements of the VERITAS™ algorithm by Hillrom which is used for beat detection. The commercial VERITAS™ algorithm is cleared by the U.S. Food & Drug Administration. Next, linear phase filtering is performed using a forth order highpass Butterworth filter with cutoff frequency of 0.5 Hz to reduce baseline wander. Finally, the signal is confined in amplitude by thresholding to ±1 mV. All beats detected by the VERITAS™ algorithm, including ectopic beats, are considered in the process of generating the ECM-images.

**Figure 1:**
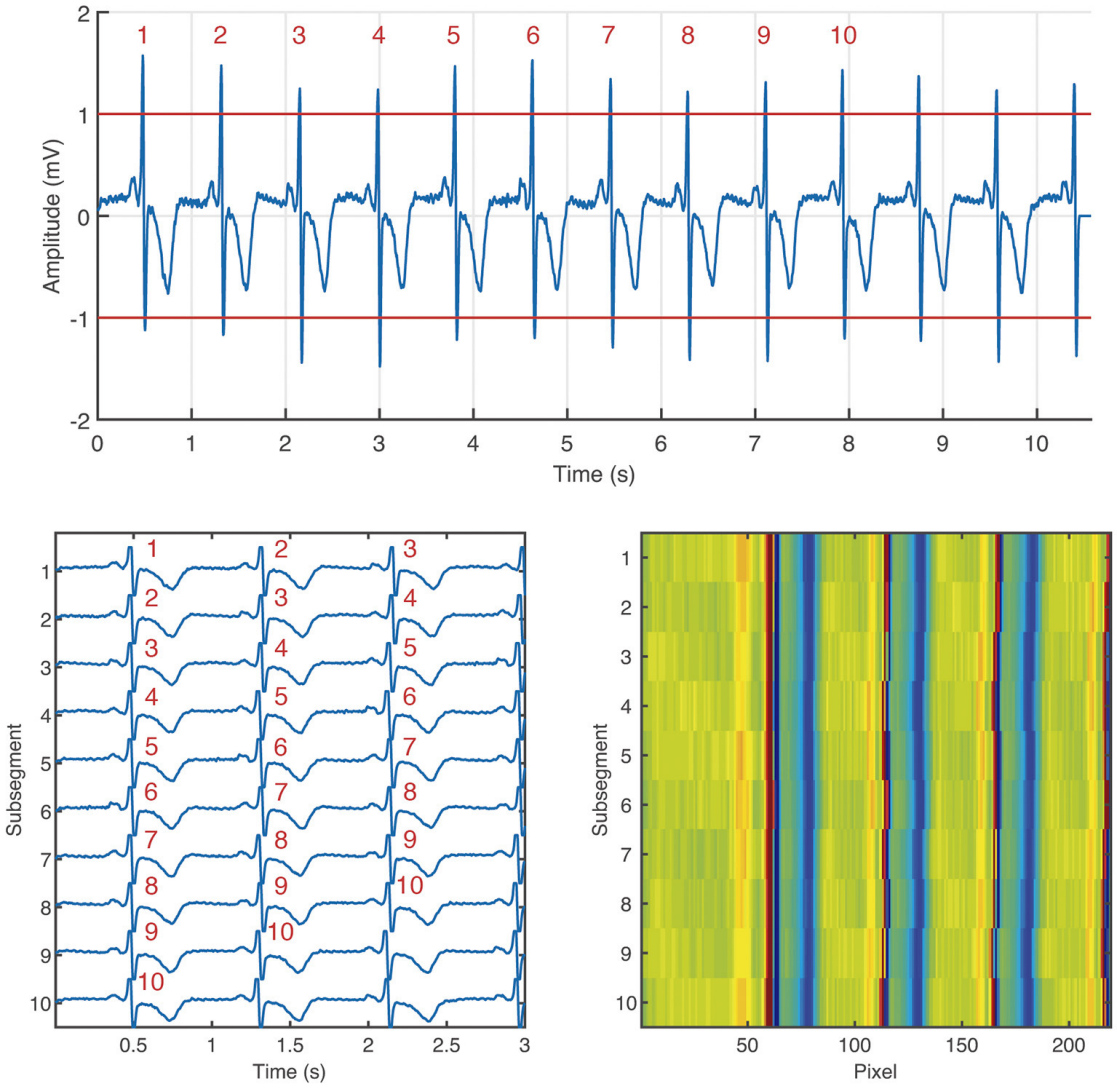
Generation of the ECM-image from an ECG segment labeled as non-AF. Segment taken from channel 1 of the record 04015 in the AFDB. The segment corresponds to the time interval [4,567, 4,578] s. On the **top**: ECG segment containing 10 beats (labeled with red numbers) plus 2.5 s after the last beat, and 0.5 s before the first beat. **Bottom left**: ECM containing the 10 subsegments aligned vertically, the corresponding beats from the ECM segment are labeled in red numbers. **Bottom right**: ECM-image.

The ECM-images are constructed from ECG segments containing 10 beats plus 2.5 s after the last detected beat and 0.5 s before the first detected beat. From each segment, 10 subsegments of 3.0 s length, partially overlapping, are derived, for which the *i*:th subsegment starts 0.5 s before the R peak of the *i*:th detected beat, 1 ≤ *i* ≤ 10. Next, the ten subsegments are aligned vertically and stored as a matrix, hereinafter the ECM. Finally, the aligned subsegments in the ECM are downsampled to: (1) 125 Hz in the interval [0, 0.5] s, (2) 50 Hz in the interval [0.5, 3.0] s. The downsampled ECM of dimensions 10 × 219 is treated as an intensity one-channel image, referred to as the ECM-image. The main motivation for the two-sections downsampling is to maintain on the left side of the ECM-image a high time resolution in the P wave interval, which is normally 120 ms before every detected beat, and removing redundant information on the right side of the ECM-image keeping only local rhythm patterns. Figure 1 depicts the process of generating an ECM-image.

The ECM-images are generated for all records and all channels of each database. A binary labeling is considered; the ECM-images are labeled as AF if 50% or more of the corresponding ECG segment was annotated as AF, otherwise it was labeled as non-AF. For the training and validation dataset, the non-AF class contains ECM-images from ECG segments from healthy subjects in normal sinus rhythm (NSR), while the AF class contains ECG segments from patients in AF. For the test dataset, the AF class contains ECM-images from patients in AF, while the non-AF class contains ECG segments from patients with other cardiac arrhythmias as well as patients in NSR, see Table 1. For the records used during training and validation, the ECM-images are generated from 5 beats overlapping windows whereas non-overlapping windows are used for the ECM-images used for testing, cf. Table 1. The former was done to augment the number of ECM-images in the training process, and the latter to mimic a clinical situation in the testing stage.

### 2.3. Convolutional Neural Network

Convolutional neural networks were proposed by Fukushima and Miyake (1982), and later boosted by LeCun et al. (1998). The structure of the network is commonly composed of a set of different layers with specific purposes. The input layer receives the information to be classified. The convolutional layers, gathering a set of sliding kernels, filter the data to extract characteristic features. The pooling layers reduce the number of features and provide robustness to variability present in the input. Activation functions such as ReLu introduce a non-linear behavior to the network to improved characterization of complex data. Fully connected layers weight all learned features, from the previous layer, providing a feature distribution to further layers. The softmax function maps the multidimensional output from the previous layer into a set of values in the range [0,1], each element in the output of the softmax layer represents the likelihood for the input to belong to each of the classes. Finally, the classification layer assigns the input to the class with the highest likelihood computed in the softmax layer. Additionally, it is a common practice to include a batch normalization block to allow higher learning rates and make the network less sensitive to initialization settings (Ioffe and Szegedy, 2015). The essence of training a neural network is to automatically tune the weights, *w*, in the kernels of the different layers so that the network correctly performs a specific task. This tuning is done following the back-propagation algorithm (LeCun et al., 1998) which is based on the error rate for each iteration.

**Figure 2:**
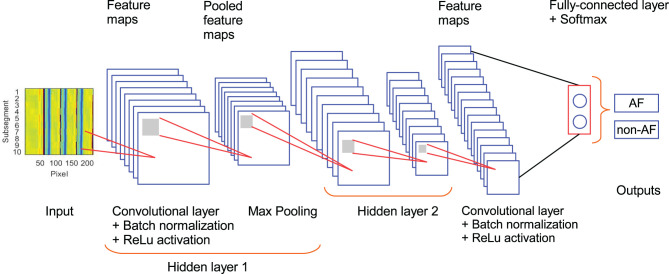
Architecture of the CNN used in the study. Hidden layer 2 contains the same set of elements as hidden layer 1.

The architecture of the CNN used in this study is described in Figure 2 and Table 2. It is composed of 3 convolutional layers, 3 batch normalization layers, 3 ReLu activation layers, 2 max pooling layers, 1 fully connected layers, 1 softmax layer, and 1 classification layer (Goodfellow et al., 2016). This architecture was selected as a trade-off between performance and number of trainable parameters during the training stage. In our CNN, the input is an ECM-image, and the output of the softmax layer provides two values representing the likelihood for the ECM-image to belong to the classes AF and non-AF, respectively. Finally, the classification layer assigns the input to the class with the highest likelihood. Three-fold cross-validation was performed to train the CNN with datasets selected manually, such that validation data were always taken from different patients than training data. During the cross-validation a subset of 80% of the ECMs created from the LTAFDB and the NSRDB was used for training and the remaining 20% was used for validation. To assure that the training subsets were balanced, we randomly excluded the surplus of the class with more ECM-images, i.e. for the training subset 1 there are ~1.6811 × 10^6^ ECM-images in the AF class, and ~1.7699 × 10^6^ in the non-AF class, therefore, 1.6811 × 10^6^ ECM-images from the non-AF class are randomly selected and used for training. This process resulted in three different trained networks with the same architecture but different learnable parameters (Net 1, Net 2, Net 3). The binary output of each CNN classifies the ECM-images either as non-AF or AF. A final classification is done by majority of votes (MoV) from the three networks (Net 1, Net 2, Net 3). In this sense, the resulting classifications from each network are contrasted with each other, and each ECM-image is placed in the class with MoV. A total of 10,217 learnable parameters are contemplated in the network. Batches of 3,000 ECM-images are used to speed up the training process (Ioffe and Szegedy, 2015), randomly shuffling the batches on each of the epochs considered. During the training and validation phase we noticed no significant improvement in accuracy after the third epoch, for this reason only 3 epochs were used for training the network. The CNN is trained using the function trainNetwork included in the Deep Learning Toolbox from MATLAB (The MathWorks, 2019). The stochastic gradient descent with momentum is used to minimize the binary cross-entropy function used as cost function, therefore, maximizing the classification accuracy. The following parameters were set: learning rate of 0.01, L2 regularization factor of 0.0001, and momentum contribution of 0.9.

**Table 2:** Properties of the CNN.

**Layer**	**Kernel size (H, L, W)**	**Stride (H, L)**	**Activations**
Input	-	-	10 ×219 ×1
Convolutiona 1	(3, 9, 10)	(1, 1)	10 ×219 ×10
Batch Normalization 1	-	-	10 ×219 ×10
ReLu activation 1	-	-	10 ×219 ×10
Max Pooling 1	(3, 3, 10)	(1, 2)	10 ×110 ×10
Convolutiona 2	(3, 9, 15)	(1, 2)	10 ×110 ×15
Batch Normalization 2	-	-	10 ×110 ×15
ReLu activation 2	-	-	10 ×110 ×15
Max Pooling 2	(3, 3, 15)	(2, 2)	5 ×55 ×15
Convolutiona 3	(2, 4, 20)	(2, 2)	3 ×28 ×20
Batch Normalization 3	-	-	3 ×28 ×20
ReLu activation 3	-	-	3 ×28 ×20
Fully Connected	-	-	1 ×1 ×2
Softmax	-	-	1 ×1 ×2
Output	-	-	1 ×1 ×2

*Letters H, L, and W stand for height, length, and width, respectively*.

*Zero padding was used on the edges on all layers*.

### 2.4. Layer-Wise Relevance Propagation

The underlying reasoning behind the classification process of CNNs is considered as a “black-box” not providing information about the basis for the decisions. To overcome the lack of transparency of the CNN, we applied the LRP methodology (Bach et al., 2015) to provide a way of explaining and interpreting the automated decisions. The overall objective of LRP is to analyze the prediction made by the CNN based on the independent contribution of all elements in the input, in our case, the input is an ECM-image and each pixel represents a single element.

Let *L*+1 be the number of layers in the CNN where the last layer is the classification layer. Then, the first relevance score, *R*^(*L*)^, is the element with the largest score in the output of the softmax layer, i.e., the *L*:th layer. The other element in the output of the softmax layer is not of interest because it is not used for classifying the input. Next, we propagate *R*^(*L*)^ to the previous layers following a set of propagation rules in order to compute the relevance scores for the lower layers such that

(1) $$\begin{array}{r} R^{\left(L\right)}=\overset{N_{L-1}}{\underset{n=1}{\sum }}R_{n}^{\left(L-1\right)}. \end{array}$$

where *N*_*L*−1_ is the number of neurons in layer *L*−1, and $R_{n}^{\left(L-1\right)}$ is the relevance score of neuron *n* in layer *L*−1. Positive values of $R_{n}^{\left(L-1\right)}$ implies that the neuron *n* contributes to the decision, whereas negative values of $R_{n}^{\left(L-1\right)}$ implies that the neuron contributes against the decision.

A relevance score $R_{n}^{\left(l\right)}$ is associated to each neuron *n* in layer *l*. The relevance score $R_{k}^{\left(l-1\right)}$ for neuron *k* in layer *l*−1, which is closer to the input layer, can be computed based on the relevance scores of the neurons in layer *l* using the following equation.

(2) $$\begin{array}{r} R_{k}^{\left(l-1\right)}=\overset{N_{l}}{\underset{n=1}{\sum }}\frac{a_{n}w_{n,k}}{\epsilon +\underset{0,k}{\sum }a_{n}w_{n,k}}R_{n}^{\left(l\right)}, \end{array}$$

where *w*_*n, k*_∈ℝ are connection weights between neuron *n* and neuron *k*, and *a*_*n*_∈ℝ is the activation of neuron *n*. The sum $\underset{0,n}{\sum }$ runs over all neurons in layer *l*−1, plus the bias neuron *w*_0, *k*_ for which *a*_0_ = 1. Equation (2) is known as the *Epsilon Rule* (LRP-ϵ) since it includes a small positive term ϵ that absorbs some negative relevance contributions to the activation of neuron *k* (Bach et al., 2015). As ϵ becomes larger only the most relevant activations survive the absorption.

**Figure 3:**
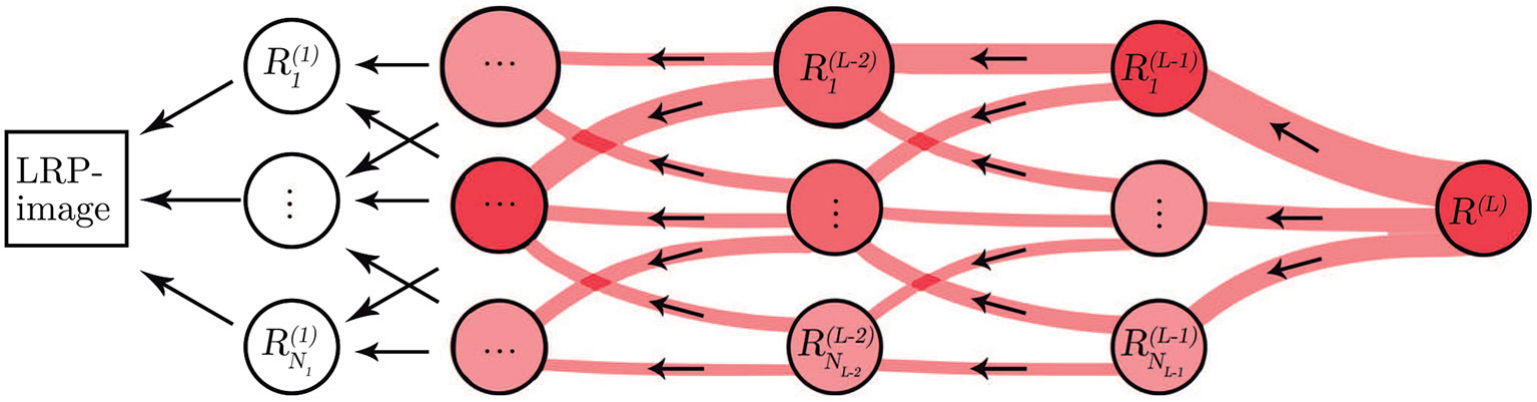
Illustration of the LRP procedure. Relevance from each neuron propagates to neurons in a lower layer.

The LRP process generates a relevance score for every pixel of the input ECM-image, these scores are collected in a matrix with same dimensions as the ECM-image, i.e., 10 × 219. The matrix provides information about how relevant a pixel was for the CNN to make the decision. In this sense, a score above zero indicates that the pixel contributed to the decision while a score below zero indicates that the pixel contributed against the decision. Then, the matrix is treated as an intensity one-channel image, hereinafter the LRP-image, for visualization, see Figure 3. To facilitate the interpretation of the LRP-image, we produced two images from it. The first one for the interval [0,0.5] s (i.e., [1,63] columns at 125 Hz, leftside-LRP) and the other from [0.5,3.0] s (i.e., [64,219] columns at 50 Hz, rightside-LRP), in accordance with the two-sections downsampling in section 2.2. These images were computed as an average from the ten rows in the LRP-image by remapping its information to the ECG segment in time domain, in other words, reversing the process for creating the ECM-images, cf. Figure 1. In this sense, we aligned the rows in the LRP-image with each other making sure that the beats present in each row are aligned. Finally, the absolute relevance was computed by averaging the aligned relevance scores in the rows. This process was made individually for each side; leftside-LRP and rightside-LRP.

### 2.5. Evaluation Metrics

The capacity of the CNN to classify the ECM-images is evaluated by determining the number of ECM-images correctly classified as AF (true positives, *TP*), the number of ECM-images correctly classified as non-AF (true negatives, *TN*), the number of ECM-images falsely classified as AF (false positives, *FP*), and the number of ECM-images falsely classified as non-AF (false negatives, *FN*). Some commonly used performance metrics that can be computed from the previously listed counts are: accuracy (*Acc*), sensitivity (*Se*), specificity (*Sp*), positive predictive value (*PPV*), F1-score (*F*1), and Matthews correlation coefficient (*Mcc*). These metrics are defined in Equations (3)–(8). The metrics in Equations (3)–(7) take values in the interval [0,1], where 1 corresponds to perfect performance. However, *Mcc* in Equation (8) takes values in the interval [−1,1]. To facilitate comparison of the *Mcc* score to the other metrics, the *Mcc* is normalized to the interval [0,1]. The *Acc* score quantifies the proportion of correct classifications among the total number of classifications. Sensitivity and specificity measure the proportion of positives that are correctly identified and the proportion of negatives that are correctly identified, respectively. The proportion of *TP* that are identified among all positive classifications (i.e., *TP*+*FP*) is captured by the *PPV* score. The harmonic mean of *PPV* and *Se* is represented by *F*1, and the *Mcc* score is a balanced measure which accounts for true and false positives and negatives.

(3) $$\begin{array}{rc} Acc & =\frac{TP+TN}{TP+TN+FP+FN}, \end{array}$$

(4) $$\begin{array}{rc} Se & =\frac{TP}{TP+FN}, \end{array}$$

(5) $$\begin{array}{rc} Sp & =\frac{TN}{TN+FP}, \end{array}$$

(6) $$\begin{array}{rc} PPV & =\frac{TP}{TP+FP}, \end{array}$$

(7) $$\begin{array}{rc} F1 & =\frac{2\times TP}{2\times TP+FP+FN}, \end{array}$$

(8) $$\begin{array}{rc} Mcc & =\frac{TP\times TN-FP\times FN}{\sqrt{\left(TP+FP\right)\left(TP+FN\right)\left(TN+FP\right)\left(TN+FN\right)}}. \end{array}$$

The EC57 standard (Association for the Advancement of Medical Instrumentation, 2012) is a guideline for testing and reporting performance results of algorithms for cardiac rhythm and ST-segment measurements. Furthermore, this standard is recognized by the U.S. Food & Drug Administration as a consensus standard for medical devices. The EC57 standard emphasizes that record-by-record results should be presented, and recommends to report the results for each channel individually. The EC57 standard also recommends to include statistics describing the performance of the detector on the entire database as a whole (gross statistics) when analyzing records where the total number of events is small. Further, the EC57 standard emphasizes the importance of reporting statistics for the number of episodes detected as well as the total duration of the episodes. The performance metrics suggested by the EC57 standard are: episode sensitivity (*Se*_*Epi*_), episode positive predictive value (*PPV*_*Epi*_), duration sensitivity (*Se*_*Dur*_), and duration positive predictive value (*PPV*_*Dur*_) defined as follow:

(9) $$\begin{array}{rc} Se_{Epi} & =\frac{TP_{Epi}}{TP_{Epi}+FN_{Epi}}, \end{array}$$

(10) $$\begin{array}{rc} PPV_{Epi} & =\frac{TP_{Epi}}{TP_{Epi}+FP_{Epi}}, \end{array}$$

(11) $$\begin{array}{rc} Se_{Dur} & =\frac{T_{AF}\cap {\hat{T}}_{AF}}{T_{AF}}, \end{array}$$

(12) $$\begin{array}{rc} PPV_{Dur} & =\frac{T_{AF}\cap {\hat{T}}_{AF}}{{\hat{T}}_{AF}}, \end{array}$$

where *TP*_*Epi*_ are the number of correctly detected AF episodes, *FN*_*Epi*_ are the number of undetected AF episodes, *FP*_*Epi*_ are the number of incorrectly detected AF episodes, *T*_*AF*_ is the total duration of AF manually annotated, and ${\hat{T}}_{AF}$ is the total duration of AF detected by the algorithm.

## 3. Results

In this section we present the classification performance of the CNN in terms of the metrics listed in Equations (3)–(8). Next, we describe the application of the LRP methodology to provide interpretability to the decision process of the CNN, showing how clinical features such as rhythm irregularity and waveforms are taken into account to make the classification. Finally, each classified ECM-image is remapped to its original time-domain, hence, labeling each sample in the ECG signal. These labels are basis for *TP*_*epi*_, *FN*_*epi*_, *FP*_*epi*_, and ${\hat{T}}_{AF}$ which are used to obtain *Se*_*Epi*_, *PPV*_*Epi*_, *Se*_*Dur*_, and *PPV*_*Dur*_.

We also investigated the performance of the network when short ECG signals from a high number of patients are used for training the network. For this purpose, we used the CINC/Physionet 2017 database (Goldberger et al., 2000). A detailed description of the database, and the resulting performance are included in the Supplementary Material.

### 3.1. Training and Validation of the CNN

Using both channels of the NSRDB and LTAFDB, 2231588 and 2105012 ECM-images were generated for non-AF and AF, respectively, and used for training and validation as described in section 2.3. For the validation subset of ECM-images, *Acc* = 91.28±0.76% (mean ± std) across the three networks was achieved. A similar score was obtained for *Mcc* but the *F*1 score was slightly lower with larger variability between Net1, Net2, and Net3. The performance achieved on the validation subsets is summarized in Table 3.

**Table 3:** ECM-images classification performance from CNN on the validation subsets from the NSRDB and LTAFDB.

	**Acc**	**Se**	**Sp**	**PPV**	***F*1**	**Mcc**
Net 1	92.12	90.57	93.37	91.70	91.13	92.03
Net 2	91.04	98.58	87.44	78.96	87.69	91.00
Net 3	90.66	92.95	87.96	90.13	91.52	90.59
Mean	91.28	94.04	89.59	86.93	90.11	91.21
Std	0.76	4.11	3.29	6.95	2.11	0.74

### 3.2. Classification Performance of the CNN

For each channel of the AFDB, 60,866 and 54,459 ECM-images were generated for non-AF and AF, respectively. This dataset was used for testing the performance of the network on each channel. Similarly, for each channel of the Arrhythmia DB, a total of 9,495 and 1,077 ECM-images were generated for non-AF and AF, respectively, and used for testing the proposed method. Lastly, the network was tested on each lead of the Monzino-AF DB for which 6,689 and 24,272 ECM-images resulted for non-AF and AF, respectively. It is worth noting that the datasets generated from the Arrhythmia DB and the Monzino-AF DB are highly unbalanced while for the AFDB there is a similar number of ECM-images for both classes.

For channel 1 of the AFDB, *Acc* = 84.84±1.20% across the three networks was obtained during testing while for channel 2, *Acc* = 87.88±3.05% was obtained. The MoV accuracy was 86.46 and 89.99% for channel 1 and channel 2, respectively, indicating a large agreement between the trained networks. Given the similar amount of ECM-images for each class for this database, *Mcc* provides similar results to *Acc*, while *F*1 resulted in a lower score due to the low number of *TP*, also reflected in the low *Se*. Table 4 summarizes the performance of the presented approach on the AFDB.

**Table 4:** ECM-images classification performance on AFDB.

	**Channel 1**						**Channel 2**					
	**Acc**	**Se**	**Sp**	**PPV**	***F*1**	**Mcc**	**Acc**	**Se**	**Sp**	**PPV**	***F*1**	**Mcc**
Net 1	86.21	73.88	97.25	96.01	83.50	86.92	88.13	82.91	92.80	91.15	86.83	88.18
Net 2	84.35	73.02	94.48	92.21	81.50	84.82	90.80	86.97	94.23	93.09	89.93	90.82
Net 3	83.96	77.37	89.87	87.23	82.00	84.02	84.71	83.82	85.50	83.80	83.81	84.66
Mean	84.84	74.76	93.87	91.82	82.34	85.25	87.88	84.56	90.84	89.35	86.86	87.88
Std	1.20	2.30	3.73	4.40	1.04	1.50	3.05	2.13	4.68	4.90	3.06	3.09
MoV	86.46	75.92	95.88	94.29	84.11	86.92	89.99	86.56	93.07	91.78	89.09	89.99

Next, we tested the network on the Arrhythmia DB for which the MoV accuracy was 79.29% and 81.33% for channel 1 and 2, respectively. It should be pointed out that for the Arrhythmia DB some non-AF ECM-images are generated from ECG segments with cardiac arrhythmias, cf. Table 1. The reduced accuracy achieved in comparison to that obtained on the AFDB is mostly due to *FP*s resulting from ECM-images in the non-AF class containing other cardiac arrhythmias. For this database, the *Mcc* score is lower than *Acc* as expected for unbalanced classes while *F*1 score is notably reduced due to a considerable number of *FP*s. Table 5 presents the performance metrics for the Arrhythmia DB.

**Table 5:** ECM-images classification performance on Arrhythmia DB.

	**Channel 1**						**Channel 2**					
	**Acc**	**Se**	**Sp**	**PPV**	***F*1**	**Mcc**	**Acc**	**Se**	**Sp**	**PPV**	**F*1***	**Mcc**
Net 1	79.78	98.98	77.60	33.39	49.93	75.22	82.79	87.56	82.25	35.88	50.90	74.43
Net 2	77.61	96.47	75.47	30.85	46.75	73.35	77.95	81.24	77.58	29.13	42.88	69.73
Net 3	75.77	98.61	73.18	29.43	45.33	72.90	77.79	87.37	76.70	29.84	44.49	71.18
Mean	77.72	98.02	75.42	31.22	47.34	73.82	79.51	85.39	78.85	31.62	46.09	71.78
Std	2.01	1.35	2.21	2.01	2.36	1.23	2.84	3.59	2.98	3.71	4.24	2.41
MoV	79.29	99.16	77.04	32.88	49.39	74.98	81.33	88.21	80.55	33.96	49.04	73.57

The results from the proprietary Monzino-AF DB show that lead II allows more accurate classification than lead I. This observation is not possible from the results from the AFDB and the Arrhythmia DB, for which channel 1 and channel 2 are undefined. Interestingly, ECM-images from lead V5 achieved the highest performance for *Acc, F*1, and *Mcc* over all other leads. Table 6 shows the MoV performance for the Monzino-AF DB. Similarly to the results from the Arrhythmia DB, the *Mcc* was lower than *Acc* due to unbalanced classes. However, *F*1 surpasses both *Acc* and *Mcc* since there are many more ECM-images in the AF class for this database. The lowest *Acc*, *F*1, and *Mcc* over all three databases are obtained for the Arrhythmia DB indicating that non-AF arrhythmias represent a significant confounding factor for the CNN when making the classification.

**Table 6:** Majority of voting ECM-image classification performance on Monzino-AF DB.

**Lead**	**I**	**II**	**III**	**V1**	**V2**	**V3**	**V4**	**V5**	**V6**
*Acc*	81.22	87.02	89.08	81.57	78.81	76.51	88.00	90.69	84.69
*Se*	80.53	88.30	89.68	77.87	75.70	73.67	88.69	92.44	85.44
*Sp*	83.70	82.37	86.90	94.98	90.09	86.83	85.48	84.33	81.99
*PPV*	94.72	94.79	96.13	98.25	96.52	95.30	95.68	95.54	94.51
*F*1	87.05	91.43	92.79	86.88	84.85	83.10	92.06	93.97	89.74
*Mcc*	78.04	82.77	85.55	80.90	77.82	75.47	84.25	86.92	80.53

### 3.3. Layer-Wise Relevance Propagation Scores

**Figure 4:**
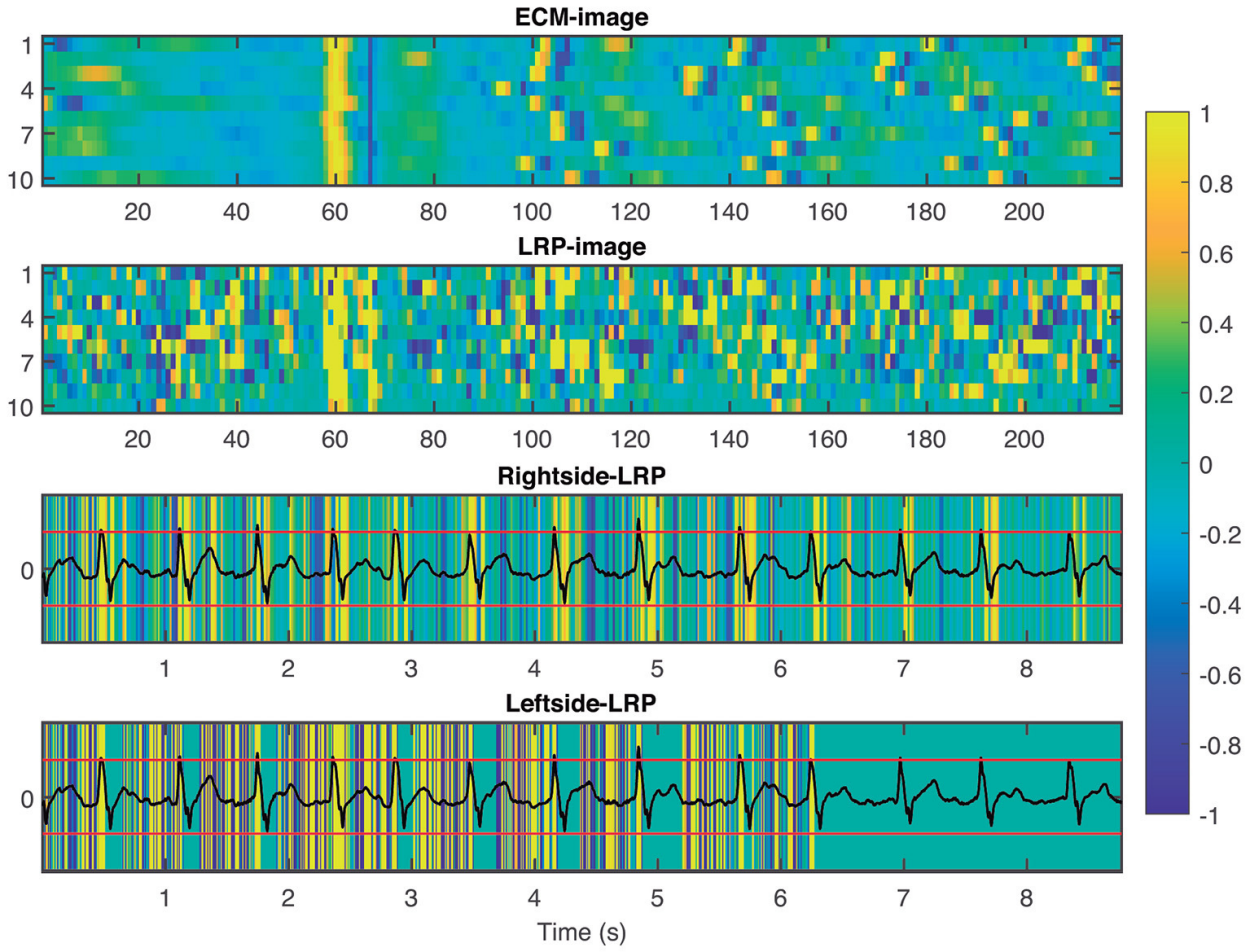
From **top** to **bottom**: ECM-image labeled as AF and correctly classified as AF, heatmap image resulting from the LRP process, rightside-LRP, and leftside-LRP. The rightside-LRP mainly highlights the detection of the QRS complexes while the leftside-LRP captures morphological information used for the classification.

**Figure 5:**
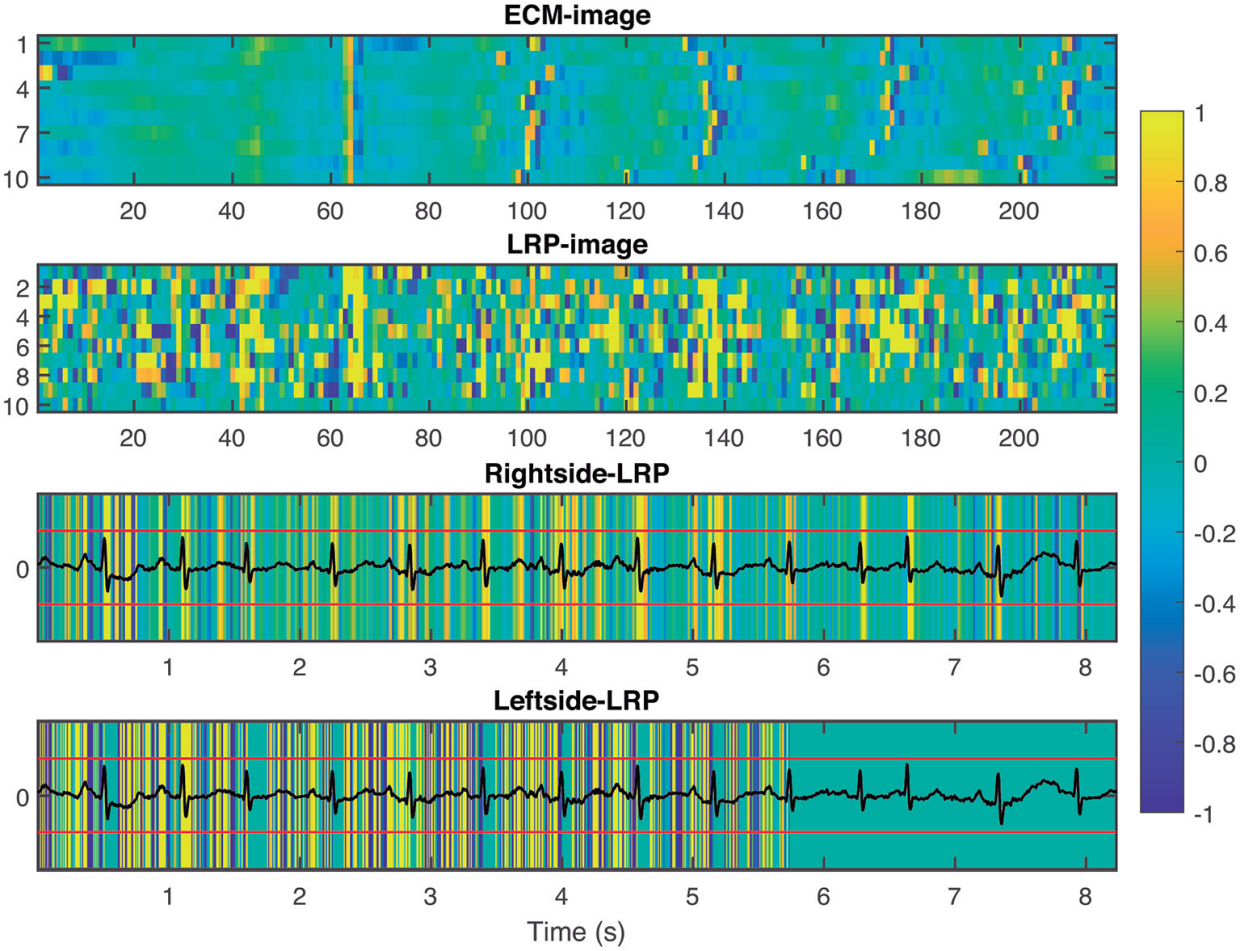
From **top** to **bottom**: ECM-image labeled as non-AF and correctly classified as non-AF, heatmap image resulting from the LRP process, rightside-LRP, and leftside-LRP. The rightside-LRP mainly highlights the detection of the QRS complexes while the leftside-LRP captures morphological information used for the classification.

**Figure 6:**
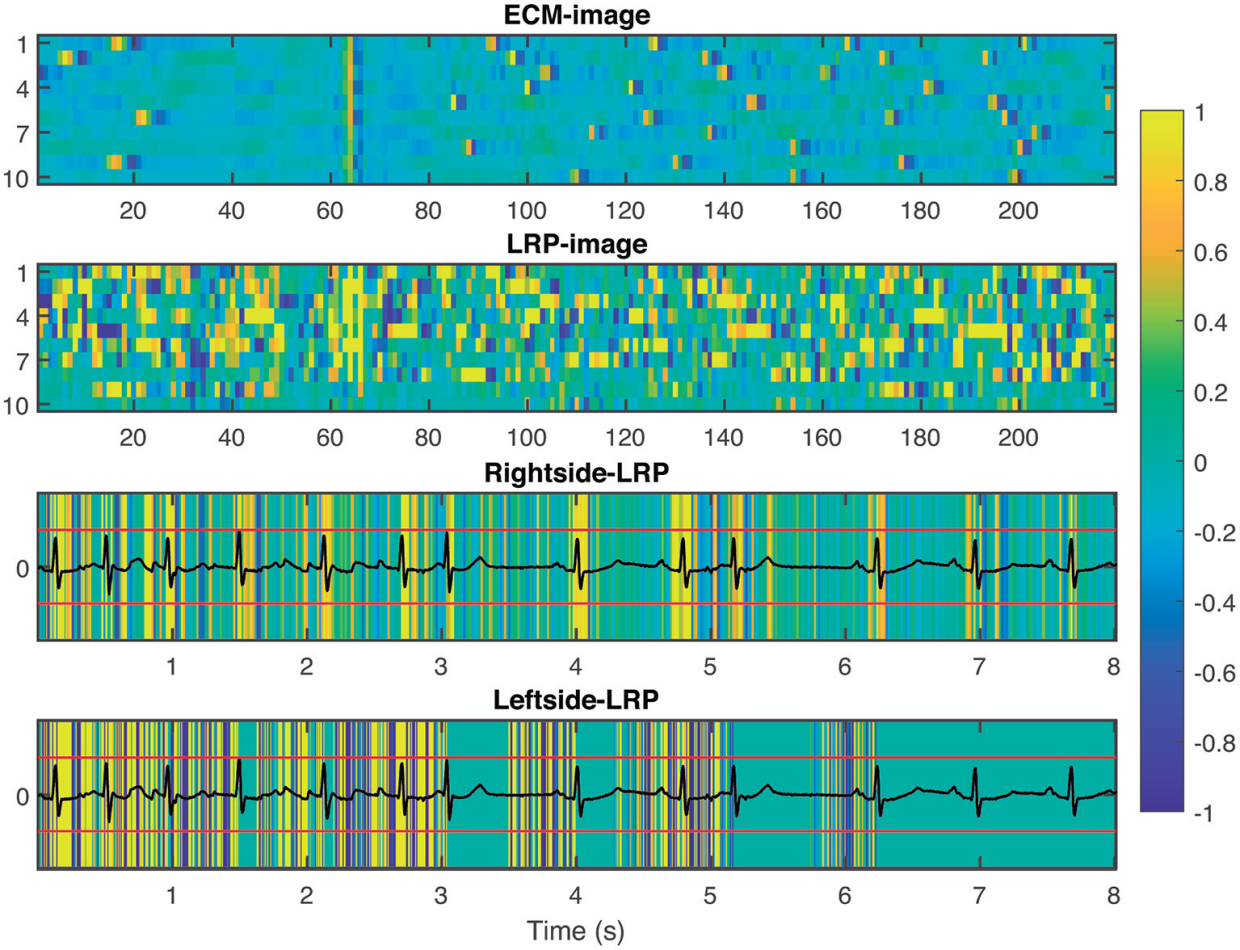
From **top** to **bottom**: ECM-image labeled as non-AF and misclassified as AF, heatmap image resulting from the LRP process, rightside-LRP, and leftside-LRP. Premature beats are interpreted as irregular rhythm by the network resulting in a FP prediction.

To provide interpretability to the decision made by the CNN, we applied the LRP technique to highlight the most relevant pixels in the ECM-image for the classification. The output of the LRP process is a matrix containing relevance scores for each pixel of the ECM-image used as input. Next, from the LRP-image, we created the leftside- and rightside-LRP images, as described in section 2.4, to facilitate interpretation. In general, these two images highlight the capacity of the CNN to extract features related to clinical concepts such as waveforms, see Figures 4–6. Figure 4 shows an example of an ECM-image labeled as AF and correctly classified as AF together with its corresponding LRP-image. The ECM-image highlights the irregularity of the rhythm as the QRS complexes from the ECG segment do not result in a regular pattern. This behavior is taken into account by the CNN to make the correct classification as shown in the rightside-LRP image where the QRS complexes are given high relevance scores. Figure 5 shows an ECM-image labeled as non-AF and correctly classified as non-AF as well as the corresponding LRP-image. In this case, the ECM-image presents a regular pattern with vertical stripes resulting from the regular rhythm present in the ECG segment. Once again, the QRS complexes are given high relevance scores, as shown in the rightside- and leftside-LRP images. Additionally, for the non-AF ECM-images, P waves are also given high relevance scores in both images. Finally, Figure 6 illustrates an ECM-image labeled as non-AF and incorrectly classified as AF. This example shows how non-AF arrhythmias is a strong confounding factor highlighting the irregularity of the QRS complexes in bigeminy episodes.

### 3.4. Performance for AF Detection

Following the EC57 standard, duration and episode gross statistics for all three testing databases are presented in Table 7. It is worth noting the high similarity between *Se*_*Dur*_ and *PPV*_*Dur*_ in Table 7 and *Se*, and *PPV* in Tables 4–6. This observation is linked to the fact that non-overlapping ECG segments of very short duration were used for creating the ECM-images, providing also a high resolution comparison with the manual annotations. Closer inspection of Table 7 shows the importance of reporting both duration and episode statistics. In the case of the AFDB we can infer that the present approach for representing the ECG segments as ECM-images enables detection of most AF episodes (high *Se*_*Epi*_) which is very important when assessing long-term ECG recordings. As for the duration sensitivity, a low *Se*_*Dur*_ score indicates that not the full duration of an AF episode was detected but only a fraction of it. On the contrary, the low *PPV*_*Epi*_ score indicates a high *FP*_*epi*_, caused by a large number of incorrectly detected AF episodes of short duration. Finally, the high *PPV*_*Dur*_ score indicates that most of the AF episodes detected by the algorithm were correct. A similar behavior is observed for the Arrhythmia DB and the Monzino-AF DB.

**Table 7:** Gross duration- and episode-performance on the different testing data bases.

	**AFDB**			**Arrhythmia DB**	
**Channel**	**1**	**2**		**1**	**2**
*Se*_*Dur*_	75.95	86.71		95.32	85.26
*PPV*_*Dur*_	93.40	89.85		31.50	31.05
*Se*_*Epi*_	96.73	97.45		98.13	90.65
*PPV*_*Epi*_	80.15	61.10		8.32	12.99
	**Monzino-AF DB**							
**Lead**	**I**	**II**	**III**	**V1**	**V2**	**V3**	**V4**	**V5**	**V6**
*Se*_*Dur*_	78.35	86.25	88.12	74.35	72.70	70.51	86.54	90.14	82.96
*PPV*_*Dur*_	93.99	93.28	95.66	97.76	95.39	94.25	94.86	94.62	93.75
*Se*_*Epi*_	94.23	98.08	86.54	86.54	82.69	82.69	90.38	92.31	86.54
*PPV*_*Epi*_	81.24	72.40	79.73	87.88	86.09	81.53	76.12	64.68	78.84

*The low PPV values for the Arrhythmia DB are due to FPs resulting from segments containing other cardiac arrhythmias*.

**Figure 7:**
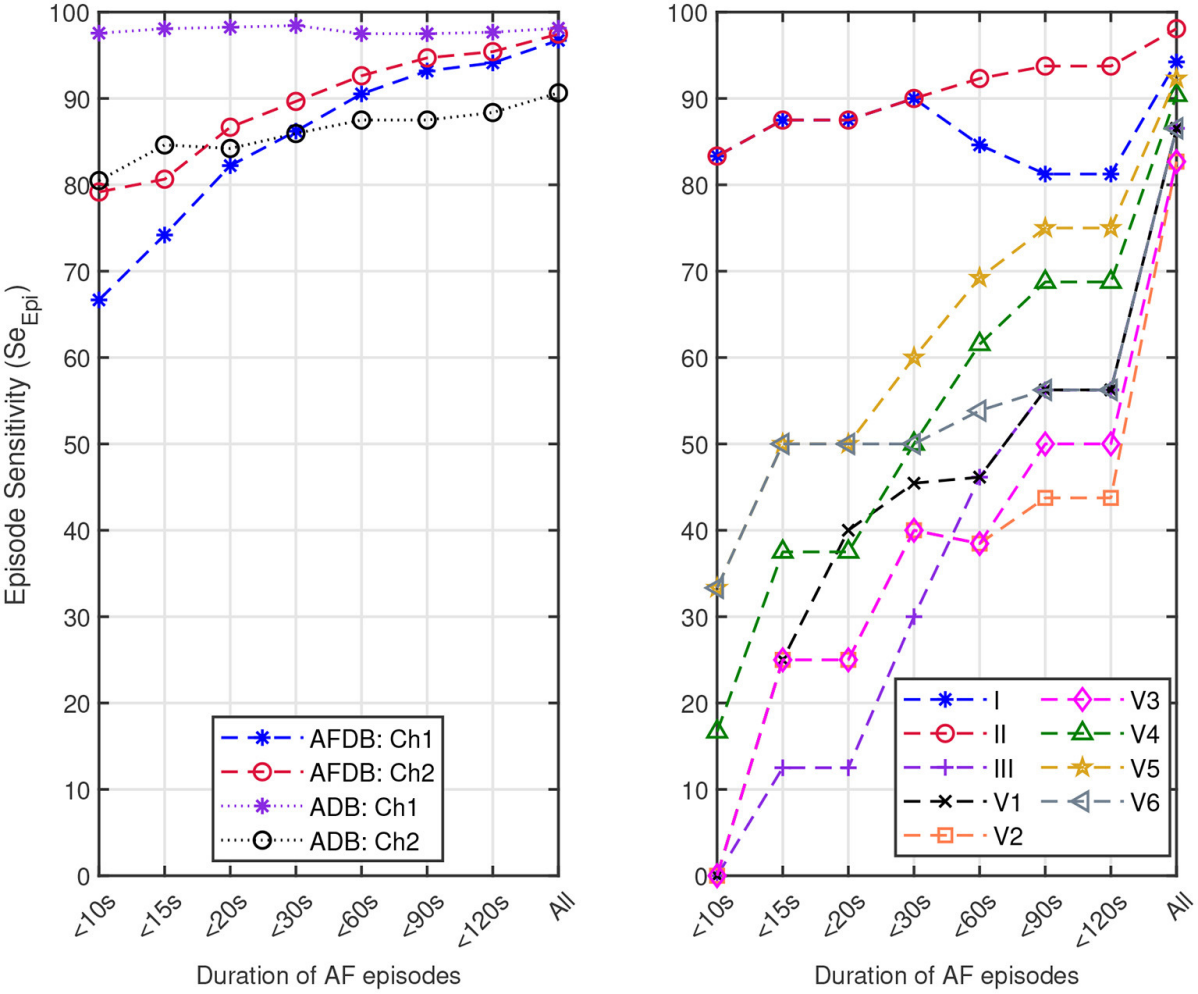
Episode sensitivity achieved on the episodes shorter than a particular duration present in: **(left)** channel 1 (Ch1) and channel 2 (Ch2) of the AFDB and Arrhythmia DB (ADB), respectively, and **(right)** leads I, II, III, V1, V2, V3, V4, V5, and V6 of the Monzino-AF DB.

We also assessed the capacity of the network for the detection of brief AF episodes. For this purpose we focus on the sensitivity achieved on episodes shorter than a particular duration. The same ECM-images generated previously were used for this analysis (cf. section 2.2). After classification, the images were remapped to their original time-domain to label each sample. From the manual annotations, Table 8 reports the number of AF episodes present in each dataset as well as the number of episodes that are shorter than 10, 15, 20, 30, 60, 90, and 120 s, respectively. Figure 7 presents *Se*_*Epi*_ achieved on brief episodes for the different testing databases. For the public databases, *Se*_*Epi*_ above 66.67% was achieved for episodes shorter than 10 s which was increasing as longer episodes were considered, and reaching a maximum above 97.45% when all episodes were studied. On the other hand, for the Monzino-AF DB only leads I and II seem to provide *Se*_*Epi*_ higher than 83.33% for episodes shorter than 10 s increasing up to 98.08% when all episodes are considered.

**Table 8:** Number of AF episodes present in the databases that are shorter than a particular duration.

**Database**	** <10*s***	** <15*s***	** <20*s***	** <30*s***	** <60*s***	** <90*s***	** <120*s***	**All**
AFDB	24	31	45	58	95	132	153	275
Arrhythmia DB	41	52	57	64	80	80	86	107
Monzino-AF DB	6	8	8	10	13	16	16	52

## 4. Discussion

In this study, we presented a new approach for AF detection making use of ECM-images and CNN. The construction of the ECM-images is based on detected beats and knowledge about ECG waveform characteristics. In the present study, a commercial algorithm that is routinely used in clinical practice was used for beat detection. All beats detected by the algorithm including ectopic beats were considered, suggesting that accurate beat detection is not critical to the performance of the method. The proposed approach combines features with clinical meaning (cf. Figures 4–6) and deep learning reaching performance accuracy above 81.33% for all testing databases when selecting the best performing channel, cf. Tables 4–6. Next, we analyzed the decision process of the CNN following the LRP methodology. The results from the LRP analysis suggest that the discriminatory features extracted by the CNN account classical non-AF characteristics such as RR interval regularity and P wave presence. Finally, we assessed the performance of the proposed methodology following the EC57 standard, first considering all AF episodes and then accounting only for brief episodes. We achieved *Se*_*Epi*_ above 97.45% when all AF episodes were considered, and above 66.67% for brief AF episodes shorter than 10 s increasing as the length of the episodes gets longer over the three testing databases, cf. Figure 7.

The ECM technique is a method to transform the ECG information into a two-dimensional representation. We applied this technique by truncating, downsampling, and shifting the ECG signal. The main advantages of using the ECM-images as input to the CNN are reduced computational complexity and improved interpretability. This was achieved by analyzing images of only 2,190 pixels created directly from the ECG signals. One drawback of this approach is that the duration of the ECG segment used to create the ECM-image does not correspond to a fixed length since they are created from a fixed number of beats. The mean ± std duration of the ECG segments generated from the testing databases is 9.55 ± 1.54 s, 10.10 ± 1.74 s, 9.24 ± 1.93 s for the AFDB, Arrhythmia DB, and the Monzino-AF DB, respectively. The ECM-images are capable of preserving morphology and rhythm characteristics of the ECG making them useful when classifying the ECM-images as AF or non-AF.

Accuracy and *F*1 score are among the most popular adopted metrics in binary classification tasks. However, when the classes in the datasets are unbalanced (i.e., the number of elements in one class is much larger than the number of elements in the other class), *Acc* and *F*1 scores tend to be inflated. Therefore, in order to address this issue, it has been suggested to report the *Mcc* score as it does not seem to be influenced by unbalanced classes (Chicco and Jurman, 2020; Butkuviene et al., 2021). Additionally, *F*1 score has the disadvantages that it varies when swapping the classes, and that it is independent from *TN* predictions (Chicco and Jurman, 2020). These two behaviors were observed for the Arrhythmia DB and the Monzino-AF DB, as these datasets are unbalanced for AF and non-AF. From the Arrhythmia DB and Monzino-AF DB results we noticed that *Acc* is not robust to unbalanced datasets resulting in higher scores than *Mcc* which handles unbalanced datasets. For the Arrhythmia DB, for which the number of elements in the non-AF is higher, the *F*1 score is very low, while it is higher for the Monzino-AF DB which has more elements in the AF class. This behavior would be the opposite if the positive class (AF) is renamed negative (non-AF) and vice versa because the *F*1 score is independent from the number of elements correctly classified as negative.

To evaluate the performance of the proposed methodology, we first tested the network on two public databases: (1) AFDB which is the most commonly used database to evaluate the performance of AF detectors, and (2) Arrhythmia DB which contains a large amount of brief AF episodes, see Table 8. Results in Tables 4, 5 highlight how non-AF arrhythmias influence the classification of ECM-images; lower *Mcc* is achieved when evaluating the Arrhythmia DB. Direct comparison of the other parameters in these two tables is not simple because the dataset of ECM-images generated from the Arrhythmia DB is highly unbalanced.

Next, the network was tested on the proprietary Monzino-AF DB to evaluate the performance on the different leads since the public databases did not provide enough information on the lead configuration. For the Monzino-AF DB the best classification was achieved using lead V5. This was most likely because high relevance was given by the network to the QRS complexes (i.e., rhythm irregularity) for making the classification leading lead V5 to better performance as this is one of the lateral leads capturing ventricular activity. It should be noted that the network was trained using 2-channels ECGs. Training the network using 12-lead ECG may have resulted in better performance for lead V1 where the atrial activity is more pronounced. Interestingly, among all leads in the Monzino-AF DB, *Se*_*Epi*_ is highest for lead II while the best *Se*_*Dur*_ is achieved on lead V5 (see Table 7). These results are consistent with the ones presented in Table 6. In this case, *Se*_*Dur*_ is analogous to the classification made by the CNN for which non-overlapping ECM-images created from short ECG segments are used as input.

The implementation of a CNN to automatically detect AF episodes from the ECM-images did not outperform manual evaluation of ECM-images for detecting AF episodes (Lee et al., 2018). In that study the authors showed that manual inspection of ECM-images provides sensitivity of 99.2%, and specificity of 99.8% when detecting AF episodes in the AFDB in contrast to the 86.7 and 93.4% achieved using the CNN in this present study for *Se*_*Dur*_ and *PPV*_*Dur*_, respectively. However, Lee et al. manually reviewed ECM-images created from the full long-term ECG recordings facilitating the detection of changes in rhythm and the presence of P waves. This represents a more time-consuming task for the reviewer. Moreover, it is important to mention that the record 04936 was excluded from the analysis made by Lee et al. because it contains a large number of paroxysmal episodes of AF. We also compared the performance of our method against the coefficient of sample entropy (COSEn) method by Lake and Moorman (2011). The COSEn is based on the RR-interval series and allows classification of short ECG segments by maximizing a decision threshold. From this analysis, we noticed that the COSEn performs with high accuracy when classifying long ECG segments. One limitation of the COSEn method is the detection of brief AF episodes. A detailed description of this analysis is presented in the Supplementary Material.

The AFDB have been used for tuning the optimal design parameters of different approaches for automatic detection of AF. For example, Dash et al. (2009) implemented a detector tuning a set of thresholds to identify AF episodes based on the randomness and variability of the RR intervals. The authors utilized a detection window of 128 beats with an average transition delay of only 18 beats, achieving *Acc* = 99.10%. Similarly, Lee et al. (2013) introduced a method based on time-varying coherence functions, for which *Acc* = 97.91% and *Acc* = 92.22% were achieved for detection windows of 128- and 12-beats, respectively. Another detector based on short RR-time series was presented by Lake and Moorman (2011), in which the coefficient of sample entropy was calculated once per hour using 12-beats, reaching *Se* = 91.00% and *Sp* = 94.00%. Approaches trained with the AFDB that assess the presence of the P-wave also allow detection of brief episodes of AF. For instance, P-wave absence was investigated using nine morphological and statistical features (Ladavich and Ghoraani, 2015). Using windows of seven beats from 20 records of the AFDB, the authors reported *Se* = 98.09% and *Sp* = 91.66%. On the contrary, Ródenas et al. (2015) and Ródenas et al. (2017) assessed the presence of the P-wave by measuring the wavelet entropy (WE) from the median TQ interval and the variability of the TQ interval series, respectively. For the former, the WE of 10 beats was used to make the detection with an average delay of 5 beats, resulting in *Acc* = 95.28%. For the latter, the variability of 15 beats was measured for detecting an AF episode with average delay of 13 beats, reaching *Acc* = 96.43%. One problem when investigating P-wave morphology is dealing with noisy records. In fact, for the studies from Ladavich and Ghoraani (2015), Ródenas et al. (2015), and Ródenas et al. (2017) manual revision of the data was needed before running the experiments.

Deep learning approaches trained and tested with subsets of the AFDB have considered different techniques. Faust et al. (2018) implemented a bidirectional long short-term memory (LSTM) network fed by sequence of 100 RR-intervals, they used data from 20 records of the AFDB for training and 10-fold cross-validation with the remaining 3 records for testing the model for which they reported *Acc* = 99.77%. The authors didn't provide the list of records used for testing so as to facilitate comparison. Similarly, Andersen et al. (2019) proposed a CNN-LSTM working with sequences of 30 RR-intervals. The model was trained and tested with the records in the AFDB (18 for testing and 5 for training, not listed). Additionally, it was also tested on the Arrhythmia DB and the NSRDB. The outputs of the CNN-LSTM model were postprocessed to reduce the number of false onsets and offsets applying a median filter to the prediction output. The performance reported on the testing subset from the AFDB was *Acc* = 97.80±0.61% while for the Arrhythmia DB they achieved *Acc* = 87.40%. It is also worth noting that the size of the data buffers used in these studies precludes detection of brief AF episodes. Further, note that the presented values of *Acc* are not directly comparable to our results, since the results were based on subsets of the AFDB.

One DL approach considering ECG segments of 5 s duration was presented by Xia et al. (2018). For this study, twenty three records of the AFDB were used for training and validation using a cross-validation approach for which subsets with proportion 9:1 were randomly selected, for which segments from one patient could be used both for training and validation (intra-patient results). Performances achieving *Acc* = 98.29% and *Acc* = 98.63% were reported for two CNNs with similar architecture but different input, respectively. He et al. (2018) utilized ECG segments containing 5 beats for which the network was trained and tested using a subset of the AFDB, records 00735, 03665, 04936, and 05091 were excluded. Following an inter-patient testing approach, the authors first balanced the number of 5 beats segments included in each class (61,924 for non-AF, and 100,612 for AF) by randomly selecting 50,000 segments for each class. Next, subsets for training and testing were selected with portion 4:1 reaching *Acc* = 98.29%. In the approach by Jin et al. (2020). ECG signals were divided into 5 s ECG segments, resulting in 60,401 and 89,659 segments for AF and non-AF, respectively. To validate the performance of the method, the authors considered both intra- and inter-patient evaluation using the AFDB. For the intra-patients experiments, they used 90% of the data for training and 10% for testing reaching *Acc* = 98.51%. For the inter-patient evaluation with *Acc* = 95.15%, 4 records selected randomly were used for testing and the rest for training. However, the authors did not mention the records used for each stage, and used unbalanced datasets for training and validation. In the study by Mousavi et al. (2020) the input was a 5 s ECG-segment including the location of the heartbeats, and waveforms present in the segment. The network was trained and tested with the AFDB, for which 66,939 and 100,483 segments were created for AF and non-AF, respectively. The authors randomly drew the same number of samples (66,939) for both AF and non-AF classes. Performance was presented for both intra- and inter-patient evaluation. For the intra-patient evaluation, 10-fold cross-validation was implemented, using 9-folds for training and 1-fold for validation, reaching *Acc* = 98.83%. For the inter-patient evaluation *Acc* = 81.54% was achieved. No report on the records used for each stage was included.

A limitation of the previously mentioned DL approaches trained, validated, and tested the network based on subsets from the same database, not mentioning the records used for each stage, and disregarded a significant amount of data for balancing the classes (Faust et al., 2018; He et al., 2018; Xia et al., 2018; Andersen et al., 2019; Jin et al., 2020; Mousavi et al., 2020). Additionally, some studies only present results for intra-patient evaluation which are likely to be inflated, as shown in Mousavi et al. (2020). From the results obtained in our study it is clear that datasets used for training and testing play an important role when evaluating the network. Results presented in Table 3 suggest that different training datasets result in slightly different validation performance while Table 4 highlights the importance of testing the system with databases not involved in the training and validation process, simulating a real clinical situation.

In this study, we created the ECM-images from short segments of ECG with the purpose of detecting brief AF episodes. To quantify the detection performance for brief AF episodes, we measured *Se*_*Epi*_, following the EC57 standard on annotated AF episodes with short duration, cf. Table 8. The results in Figure 7 show that *Se*_*Epi*_ improves above 80% as the length of the AF episodes is greater than 15 s. The ability to detect brief AF episodes, i.e., high *Se*_*Epi*_, is important especially when analyzing signals coming from long-term recordings, as the automatic detection of AF represents a powerful guidance for physicians when manually assessing such records to provide a more accurate and faster diagnosis. In this sense, the methodology proposed in this study is capable of detecting 79.17, 97.56, and 83.33% of the AF episodes shorter than 10 s present in the AFDB, the Arrhythmia DB, and the Monzino-AF DB, respectively, cf. Figure 7. For the Monzino-AF DB, the best performance was achieved on leads I and II. One possible explanation is perhaps that channels in the LTAFDB and the NSRDB very likely correspond to leads I and II, and therefore the network is more sensitive to the characteristic waveforms of these leads. It is important to note that there are only very few brief episodes in the Monzino-AF DB, therefore, any misclassification has an important impact on the sensitivity metric. The resulting *Se*_*Epi*_ increases as the duration of the AF episodes that are considered for the analysis increases reaching its maximum value on all three testing databases above 97.45% when all AF episodes are considered, cf. Figure 7. It is worth mentioning that these brief AF episodes are detected in long-term ECG recordings of at least 30 min, cf. Table 1.

Many techniques have been proposed to address the complex interpretability of DL approaches such as CNN. Some proposals provide explanations by integrating a large number of local gradient estimates (Smilkov et al., 2017; Sundararajan et al., 2017). Other techniques accounts for a coarser estimation of the effect from a patch-like perturbation (Zeiler and Fergus, 2014; Zintgraf et al., 2017). Further methods involve the optimization of some local surrogate model (Ribeiro et al., 2016), or of the explanation itself (Fong and Vedaldi, 2017). Such techniques involve multiple neural network evaluations, resulting in computationally expensive processes (Samek et al., 2019). To the best of our knowledge, only two studies in the literature have attempt to present an interpretation of the extracted features from the ECG signals that were considered for the detection of AF (Jin et al., 2020; Mousavi et al., 2020). Jin et al. used attention mechanism to explore the impact of ECG segments at different times on the final prediction, illustrating the most relevant segments of the ECG signal used as input, while Mousavi et al. provided an empirical interpretation of the network, based on hierarchical attention, showing the important waveforms of the ECG signal used to make the predictions by considering the weights obtained for the different inputs. In this study, we applied the LRP technique (Bach et al., 2015) to provide fast and reliable explanation as this technique operates by simply propagating the prediction backwards in the neural network following a set of propagation rules, cf. Figure 3. Another advantage of the LRP is the one single parameter that needs to be tuned, i.e. ϵ. In our experiments, we set ϵ = 1 as a good balance to remove noise elements in the explanation and keep only the most relevant features (Bach et al., 2015). The output from the LRP is a matrix of relevance scores which can be treated as an intensity one-channel image, i.e. LRP-image, showing how important a particular pixel is for the classification. The goal of this analysis is to provide enough evidence to support the CNN classifications, linking the most relevant characteristics from the ECM-image to a clinical interpretation from a machine learning point of view. The rightside-LRP shows that this area in the ECM-images was used by the CNN to extract rhythm characteristics highlighting mainly the QRS complexes of the segment. On the other hand, the leftside-LRP highlights different waveforms in the 0.5 s before each heartbeat. It can be seen that positive relevance is given for QRS complex and P wave while T wave from the previous heartbeat (if present in the 0.5 s before the QRS complex) was given negative relevance, cf. Figure 5. These results are in line with the motivation we used for the two-section downsampling considered when creating the ECM-images; the left side was intended to preserve morphology information while the right side to capture rhythm information. Finally, it is also shown how non-AF arrhythmias and changes in the morphology of the ECG are a strong confounding factors for the network, cf. Figure 6 and Table 5. The results of this analysis suggests that the CNN has been trained to take into account clinical features of AF, like P-wave absence and rhythm irregularity, for the classification.

## 5. Limitations

The CNN was trained on data from a limited number of patients, c.f. Table 1. Increasing the training set may improve the generalizability and hence the performance in other ECG databases. Additionally, only a small number of brief AF episodes was investigated, c.f. Table 8, and the results need to be verified in a larger study population. The proposed methodology relies on automatically detected beats provided by a commercial algorithm. It should be emphasized that accuracy of the beat detector may influence the results.

## 6. Conclusion

In this study, we have developed a deep learning approach for detection of brief AF episodes based on ECM-images and CNN. The compact two-dimensional representation of the ECM, preserving morphological and rhythm characteristics of the ECG, allows automatic detection of brief AF episodes. To reduce the chances of overfitting, we tested the proposed method on three databases not used for training: MIT-BIH Atrial Fibrillation, MITH-BIH Arrhythmia, and the Monzino-AF. The ability of the system to detect such brief episodes was assessed by computing *Se*_*Epi*_. Results showed that *Se*_*Epi*_>80.65% is achieved when episodes shorter than 15 s are considered while *Se*_*Epi*_>89.66% is reached when accounting for episodes shorter than 30 s. Finally, the LRP analysis showed that the CNN takes clinical features, such as RR irregularity and P wave absence, into account to classify AF episodes.

## Data Availability Statement

Publicly available datasets were analyzed in this study. This data can be found here: https://physionet.org/about/database/.

## Author Contributions

RS-M performed the experiments, data analyses, and wrote the manuscript. JB, NM, and FS supervised the project. All authors contributed to manuscript revision, approved the submitted version, and conceived and designed the study.

## Conflict of Interest

RS-M, JB, and NM were employed by the company Mortara Instrument Europe s.r.l. The remaining author declares that the research was conducted in the absence of any commercial or financial relationships that could be construed as a potential conflict of interest.

## Publisher's Note

All claims expressed in this article are solely those of the authors and do not necessarily represent those of their affiliated organizations, or those of the publisher, the editors and the reviewers. Any product that may be evaluated in this article, or claim that may be made by its manufacturer, is not guaranteed or endorsed by the publisher.
