# Supplementary material for: Detection of Brief Episodes of Atrial Fibrillation Based on Electrocardiomatrix and Convolutional Neural Network
Source: Front Physiol. 2021 Aug 25;12:673819. doi: 10.3389/fphys.2021.673819 (PMC8424003; doi:10.3389/fphys.2021.673819)
Supplement: Supplementary file 1 [file Data_Sheet_1.PDF]

Supplementary Material

1 RESULTS FROM THE CINC/PHYSIONET 2017

The CINC/Challenge2017 database contains 8528 single lead ECG recordings lasting from 9 to over 60 seconds. The signals were collected using the AliveCor device with a sampling frequency of 300 Hz and have been band pass filtered by the AliveCor device. The recordings in the database are grouped in four rhythm classes: 5076 Normal, 758 AF, 2415 Other, and 279 Noisy. We generated ECM-images from each recording and placed them into two classes: non-AF (including Normal, Other, and Noisy recordings), and AF (including only AF recordings). We have performed two additional tests using the CINC/Challenge2017 dataset:

1) Testing the CNN that was trained with the NSRDB and the LTAFDB on the CINC/Challenge2017 dataset. For this experiment, a total of 25105 and 3108 ECM-images were generated for the non-AF and AF class, respectively, using no overlapping segments to generate the images. We obtained the following classification results: $Acc = 84.78$, $Se = 90.67$, $Sp = 84.05$, $PPV = 41.31$, $F1 = 56.76$, $Mcc = 77.32$ that are similar to the results for the Arrhythmia DB (cf Table 5). Next, we present the detection performance following the EC57 standard: $Se_{Dur} = 87.99$, $PPV_{Dur} = 64.67$, $Se_{Epi} = 94.46$, $PPV_{Epi} = 44.18$, (cf Table 7 for Arrhythmia DB). These results suggest that the CNN can be generalized to other databases with larger number of patients. Additionally, the results suggest that hand-held recordings can be classified with similar performance as recordings from long-term monitoring.

2) Training the CNN with the CINC/Challenge 2017 dataset, and testing on channel 1 of the AFDB. For this experiment, a total of 28721 and 5810 ECM-images were generated for the non-AF and AF class, respectively, using 5-beat overlapping segments to generate the images. We obtained the following classification performance: $Acc = 84.31$, $Se = 85.40$, $Sp = 83.35$, $PPV = 82.11$, $F1 = 83.72$, $Mcc = 84.33$. These results are comparable to the results achieved when NSRDB and the LTAFDB were used for training (cf. Table 4). Note that the ECM-images in the non-AF class for the training process in this experiment contains not only ECM-images from healthy NSR but also recordings containing other arrhythmias and noise. This suggest that the network is capable of learning features that are characteristic of AF. Finally, we also evaluated the performance following the EC57 standard $Se_{Dur} = 84.45$, $PPV_{Dur} = 80.12$, $Se_{Epi} = 100.00$, $PPV_{Epi} = 55.79$, (cf. Table 7 for AFDB). These results indicate that increasing the number of patients in the training phase improves the sensitivity of the detector. This might be due to the fact that more beat morphologies are taken into account when training the network leading to a higher number of TP. On the other hand, Sp and PPV are penalized because the number of FP increases.

2 COEFFICIENT OF SAMPLE ENTROPY FOR DETECTION OF BRIEF AF

Following the methodology by Lake & Moorman, we computed the coefficient of sample entropy (COSEn) for consecutive segments of 13 beats from the the NSRDB and the LTAFDB. The segments were labeled as AF if half or more of the beats were annotated as AF, and non-AF otherwise. Using these two databases as the training set, the optimal threshold ($th = -1.45$) of COSEn was determined by maximizing the accuracy of the classification in the training set. We tested the method on three databases: AFDB, Arrhythmia DB, and Monzino-AF DB (cf. Section 2.1 in the manuscript). The accuracy achieved when classifying the ECG segments was 91.36%, 79.85%, and 90.35%, for the AFDB, the Arrhythmia DB, and the Monzino-AF DB, respectively. Once the segments were classified, they were remapped to their original time-domain, labeling each sample in the ECG signal. Finally, performance for brief AF episodes (cf. Table 8 in the

manuscript) was tested following the EC57 standard. A comparison with best performance achieved using the ECM-images and CNN is shown in Figure S1. Similar performance is achieved for the AFDB. However, when non-AF arrhythmias are present (Arrhythmia DB), or when few brief AF episodes are present (Monzino-AF DB) the ECM-images allows higher Se_{Epi} than the COSEn. These results indicates that morphological information improves AF-detection for brief episodes.

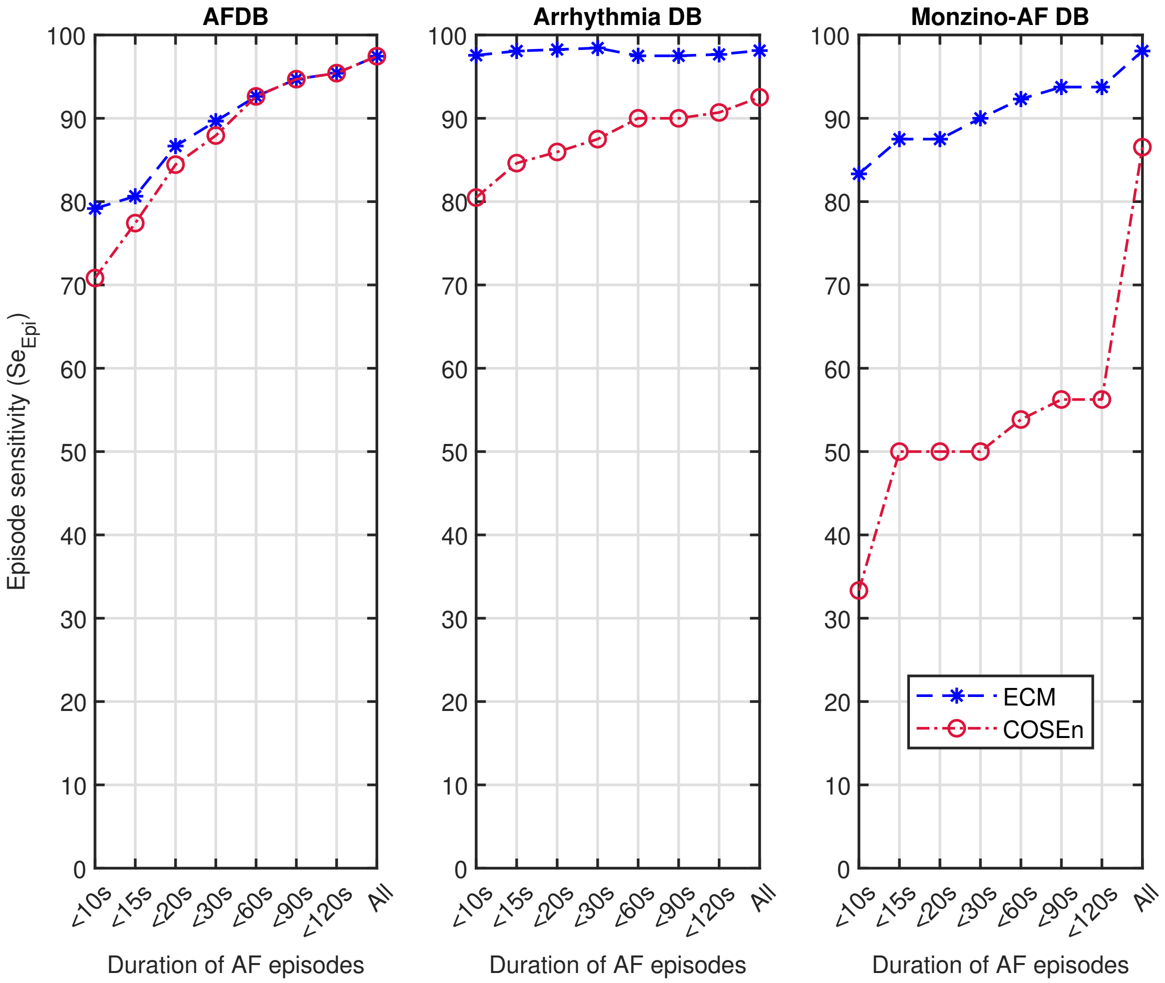

Figure S1. Episode sensitivity achieved on the testing databases. The best performance achieved using the ECM-images and the COSEn in blue and red, respectively.

3 RECORD BY RECORD PERFORMANCE

The EC57 standard Association for the Advancement of Medical Instrumentation (2012) is a guideline for testing and reporting performance results of algorithms for cardiac rhythm and ST-segment measurements. This standard emphasizes that record-by-record results should be presented. It also recommends to report the results for each channel individually. The performance metrics suggested by the EC57 standard are: episode sensitivity (Se_{Epi}), episode positive predictive value (PPV_{Epi}), duration sensitivity (Se_{Dur}), and duration positive predictive value (PPV_{Dur}). Additionally, we also report Acc, Se, Sp, PPV, $F1$, and Mcc in a record-by-record fashion for each channel. We reserved the record-by-record results only for the AFDB as it is the most common database used to assess the performance of AF detectors.

3.1 Classification performance

The performance for the classification of the ECM-images from the AFDB is presented in record-by-record fashion with average and gross statistics in Table S1.

Table S1. ECM images classification performance from CNN on AFDB in record-by-record fashion.

	channel 1						channel 2					
Record	Acc	Se	Sp	PPV	$F1$	Mcc	Acc	Se	Sp	PPV	$F1$	Mcc
n04015	97.06	100.00	97.02	28.11	43.88	76.11	93.61	100.00	93.53	15.25	26.46	68.88
n04043	89.46	98.14	86.87	69.07	81.08	88.14	95.57	97.04	95.13	85.62	90.97	94.18
n04048	99.68	98.82	99.69	87.50	92.82	96.42	99.75	95.29	99.85	93.10	94.19	97.03
n04126	96.53	99.42	96.29	69.25	81.63	90.69	83.35	98.54	82.07	31.58	47.84	75.17
n04746	97.02	95.36	99.83	99.90	97.58	96.94	99.88	99.87	99.89	99.94	99.90	99.87
n04908	99.49	97.98	99.66	97.08	97.53	98.62	96.91	99.38	96.63	77.04	86.80	92.98
n04936	89.05	87.53	98.55	99.74	93.24	84.60	94.16	96.31	80.68	96.89	96.60	87.92
n05091	99.28	90.00	99.31	25.71	40.00	73.95	99.89	100.00	99.89	71.43	83.33	92.24
n05121	83.41	76.21	97.93	98.67	85.99	84.92	88.13	97.15	69.95	86.69	91.62	86.42
n05261	99.03	100.00	99.02	66.91	80.18	90.70	90.58	100.00	90.39	17.17	29.31	69.70
n06426	95.13	95.89	71.59	99.05	97.45	74.32	92.50	93.20	71.02	99.01	96.01	69.71
n06453	98.59	93.02	98.66	46.51	62.02	82.62	98.31	90.70	98.40	41.49	56.93	80.36
n06995	88.89	85.61	92.34	92.16	88.76	89.01	83.36	96.56	69.48	76.89	85.61	84.47
n07162	73.46	73.46	NaN	100.00	84.70	NaN	58.97	58.97	NaN	100.00	74.19	NaN
n07859	60.14	60.14	NaN	100.00	75.11	NaN	96.58	96.58	NaN	100.00	98.26	NaN
n07879	99.40	99.98	98.10	99.16	99.57	99.29	99.48	99.25	100.00	100.00	99.63	99.40
n07910	97.70	97.25	97.83	93.09	95.13	96.83	98.15	97.94	98.21	94.27	96.07	97.44
n08215	42.43	25.45	100.00	100.00	40.58	63.43	92.43	99.74	67.66	91.27	95.32	88.93
n08219	85.08	96.56	81.57	61.62	75.23	84.33	94.22	96.07	93.65	82.27	88.64	92.63
n08378	52.06	21.98	97.87	94.03	35.63	63.95	50.90	20.04	97.87	93.49	33.01	63.06
n08405	64.35	53.87	99.93	99.96	70.01	72.88	78.36	72.02	99.85	99.94	83.72	80.34
n08434	96.80	100.00	96.61	63.89	77.97	89.28	98.67	76.96	99.97	99.44	86.76	93.43
n08455	93.39	91.18	100.00	100.00	95.38	92.48	84.16	78.87	99.93	99.97	88.17	84.75
mean	86.85	84.25	95.65	82.24	77.89	85.21	89.91	89.59	90.67	80.55	79.54	85.66
std	16.74	22.95	7.20	23.72	20.28	10.82	12.66	18.56	11.85	27.17	23.48	11.01
Gross	86.46	75.92	95.88	94.29	84.11	86.92	89.99	86.56	93.07	91.78	89.09	89.99

3.2 Detection performance

Duration and episode statistics for the AFDB are presented in record-by-record fashion in Tables S2-S3.

Table S2. Duration- and episode-performance from CNN on channel 1 of AFDB in record-by-record fashion.

Record	Anno_Ep	Pred_Ep	TPs	TPp	FP	FN	F_Offsets	Rec_Dur	Anno_Dur	Pred_Dur	Overlap_Dur	Se_{Dur}	PPV_{Dur}	Se_{Epi}	PPV_{Epi}
n04015	7	71	6	6	65	1	0	36823.00	237.30	1166.10	234.00	98.63	20.07	85.71	8.45
n04043	82	335	82	99	236	0	17	36823.00	7932.80	12307.10	7834.50	98.76	63.66	100.00	29.55
n04048	7	13	7	7	6	0	0	36823.00	360.80	462.70	350.70	97.19	75.78	100.00	53.85
n04126	7	121	7	9	112	0	2	36823.00	1378.00	2589.50	1361.20	98.78	52.57	100.00	7.44
n04746	5	125	3	123	2	2	120	36823.00	19553.00	18834.20	18805.10	96.17	99.85	60.00	98.40
n04908	4	31	4	13	18	0	9	36823.00	3336.10	3446.60	3294.70	98.76	95.59	100.00	41.94
n04936	36	364	35	362	2	1	328	36823.00	29951.70	26758.80	26598.40	88.80	99.40	97.22	99.45
n05091	8	26	5	5	21	3	0	36823.00	86.80	347.20	67.50	77.73	19.43	62.50	19.23
n05121	15	464	14	455	9	1	441	36823.00	23438.70	19065.90	18787.40	80.16	98.54	93.33	98.06
n05261	11	40	11	11	29	0	0	36823.00	479.50	846.70	476.80	99.44	56.31	100.00	27.50
n06426	24	202	24	200	2	0	176	36823.00	35325.70	34549.20	34155.20	96.69	98.86	100.00	99.01
n06453	6	30	6	9	21	0	3	33300.00	371.30	819.00	345.10	92.96	42.14	100.00	30.00
n06995	4	460	4	300	160	0	296	36823.00	17370.30	17016.40	15298.10	88.07	89.90	100.00	65.22
n07162	1	684	1	684	0	0	683	36823.00	36822.30	28349.80	28349.80	76.99	100.00	100.00	100.00
n07859	1	1285	1	1285	0	0	1284	36823.00	36822.90	25302.80	25302.80	68.71	100.00	100.00	100.00
n07879	1	31	1	2	29	0	1	36823.00	22209.60	22523.40	22206.80	99.99	98.59	100.00	6.45
n07910	4	69	4	19	50	0	15	36823.00	6355.20	6994.80	6245.30	98.27	89.28	100.00	27.54
n08215	1	471	1	471	0	0	470	36823.00	29724.20	7844.20	7843.80	26.39	100.00	100.00	100.00
n08219	39	448	39	66	382	0	27	36823.00	7948.90	13613.00	7707.90	96.97	56.62	100.00	14.73
n08378	5	160	4	148	12	1	144	36823.00	20643.60	4525.90	4318.70	20.92	95.42	80.00	92.50
n08405	2	441	2	440	1	0	438	36823.00	26589.50	14333.10	14325.50	53.88	99.95	100.00	99.77
n08434	3	71	3	3	68	0	0	36823.00	1423.70	2633.20	1423.70	100.00	54.07	100.00	4.23
n08455	2	228	2	228	0	0	226	36823.00	25471.00	23392.00	23392.00	91.84	100.00	100.00	100.00
Gross	275	6170	266	4945	1225	9	4680	843406.70	353832.90	287721.80	268725.00	75.95	93.40	96.73	80.15

Table S3. Duration- and episode-performance from CNN on channel 2 of AFDB in record-by-record fashion.

Record	Anno_Ep	Pred_Ep	TPs	TPp	FP	FN	F_Offsets	Rec_Dur	Anno_Dur	Pred_Dur	Overlap_Dur	Se_{Dur}	PPV_{Dur}	Se_{Epi}	PPV_{Epi}
n04015	7	93	7	6	87	0	0	36823.00	237.30	2283.90	235.70	99.34	10.32	100.00	6.45
n04043	82	169	81	98	71	1	17	36823.00	7932.80	9347.10	7731.20	97.46	82.71	98.78	57.99
n04048	7	13	7	9	4	0	2	36823.00	360.80	403.90	344.60	95.50	85.31	100.00	69.23
n04126	7	472	7	11	461	0	4	36823.00	1378.00	7967.50	1358.50	98.58	17.05	100.00	2.33
n04746	5	7	3	7	0	2	4	36823.00	19553.00	19538.00	19524.50	99.85	99.93	60.00	100.00
n04908	4	105	4	8	97	0	4	36823.00	3336.10	4644.40	3325.50	99.68	71.60	100.00	7.62
n04936	36	173	36	108	65	0	74	36823.00	29951.70	30418.80	28917.30	96.55	95.06	100.00	62.43
n05091	8	6	5	5	1	3	0	36823.00	86.80	102.80	72.40	83.45	70.47	62.50	83.33
n05121	15	304	15	78	226	0	67	36823.00	23438.70	27179.70	22811.00	97.32	83.93	100.00	25.66
n05261	11	229	11	11	218	0	0	36823.00	479.50	4076.00	478.60	99.80	11.74	100.00	4.80
n06426	24	314	24	313	1	0	289	36823.00	35325.70	33891.30	33490.60	94.81	98.82	100.00	99.68
n06453	6	32	6	9	23	0	3	33300.00	371.30	892.50	339.50	91.45	38.04	100.00	28.13
n06995	4	585	4	79	506	0	75	36823.00	17370.30	23855.20	16975.90	97.73	71.16	100.00	13.50
n07162	1	778	1	778	0	0	777	36823.00	36822.30	23244.30	23244.30	63.13	100.00	100.00	100.00
n07859	1	195	1	195	0	0	194	36823.00	36822.90	36000.20	36000.20	97.77	100.00	100.00	100.00
n07879	1	29	1	29	0	0	28	36823.00	22209.60	22093.00	22092.20	99.47	100.00	100.00	100.00
n07910	4	54	4	19	35	0	15	36823.00	6355.20	6855.50	6266.40	98.60	91.41	100.00	35.19
n08215	1	156	1	7	149	0	6	36823.00	29724.20	32230.70	29665.90	99.80	92.04	100.00	4.49
n08219	39	272	39	69	203	0	30	36823.00	7948.90	9908.30	7665.60	96.44	77.37	100.00	25.37
n08378	5	244	4	228	16	1	224	36823.00	20643.60	4454.40	4232.80	20.50	95.03	80.00	93.44
n08405	2	704	2	703	1	0	701	36823.00	26589.50	19922.80	19909.10	74.88	99.93	100.00	99.86
n08434	3	44	3	43	1	0	40	36823.00	1423.70	1197.10	1180.50	82.92	98.62	100.00	97.73
n08455	2	590	2	589	1	0	587	36823.00	25471.00	20939.50	20931.30	82.18	99.96	100.00	99.83
Gross	275	5568	268	3402	2166	7	3141	843406.70	353832.90	341446.60	306793.60	86.71	89.85	97.45	61.10

4 ADDITIONAL ECM-IMAGE

The Figure S1 shows an ECM-image labeled as non-AF and correctly classified as non-AF as well as the corresponding LRP-image. The pattern in the ECM-image results from trigeminy rhythm present in the ECG segment. The QRS complexes are given high relevance scores, as shown in the rightside-LRP image.

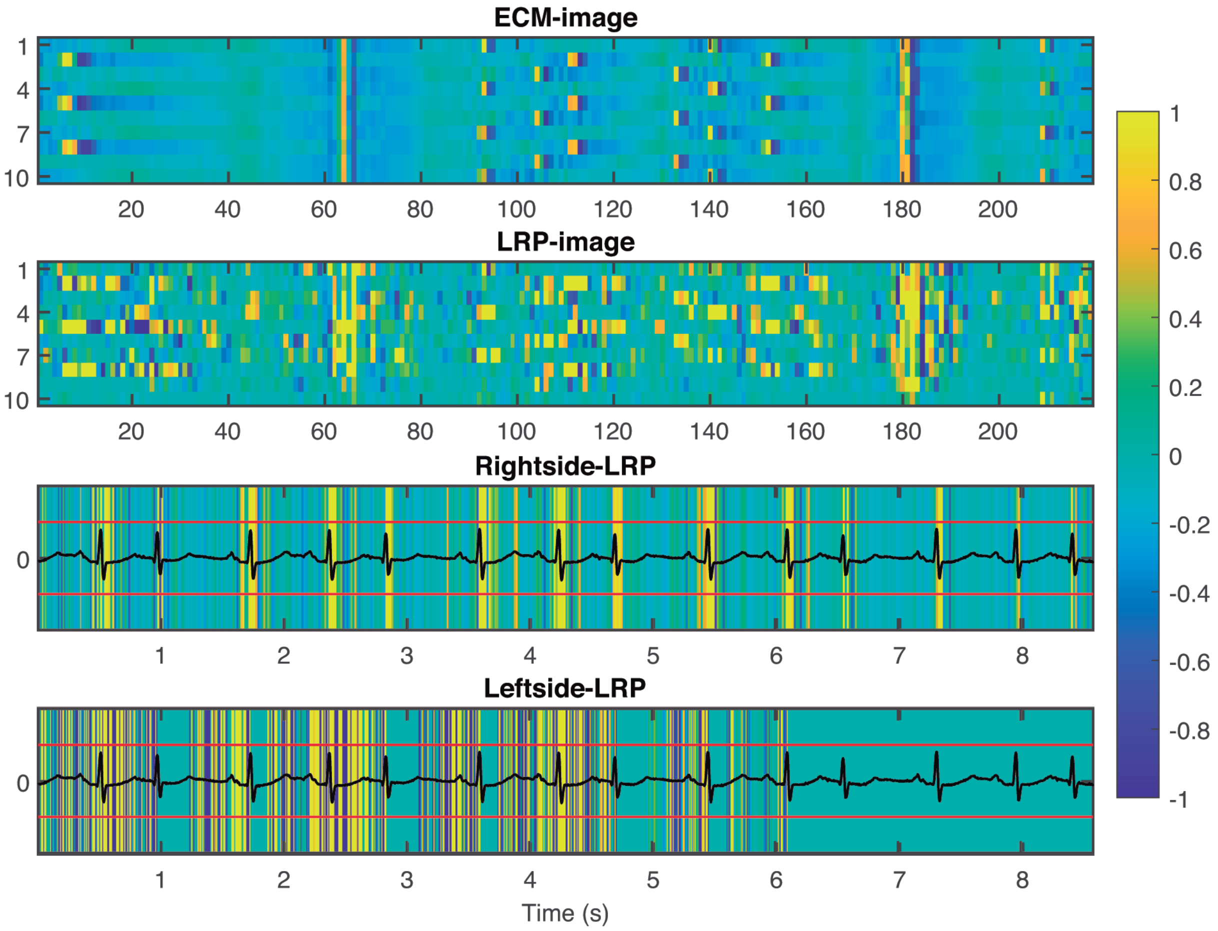

Figure S2. From top to bottom: ECM-image labeled as non-AF and correctly classified as non-AF, heatmap image resulting from the LRP process, rightside-LRP, and leftside-LRP. The rightside-LRP mainly highlights the detection of the QRS complexes while the leftside-LRP captures morphological information used for the classification.

REFERENCES

Association for the Advancement of Medical Instrumentation (2012). *ANSI/AAMI EC57:2012*. Arlington, Virginia, United States
